# Assembly patterns of soil‐dwelling lichens after glacier retreat in the European Alps

**DOI:** 10.1111/jbi.12970

**Published:** 2017-02-23

**Authors:** Juri Nascimbene, Helmut Mayrhofer, Matteo Dainese, Peter Othmar Bilovitz

**Affiliations:** ^1^ Department of BiologicalGeological and Environmental SciencesUniversity of BolognaI‐40126BolognaItaly; ^2^ Institute of Plant SciencesNAWI GrazUniversity of Graz8010GrazAustria; ^3^ Department of Animal Ecology and Tropical Biology, BiocenterUniversity of Würzburg97074WürzburgGermany

**Keywords:** dispersal traits, glacier forelands, photobiont type, primary succession, spatial‐temporal patterns, species accumulation, species richness and composition, trait selection, β‐diversity

## Abstract

**Aim:**

To assess the spatial‐temporal dynamics of primary succession following deglaciation in soil‐dwelling lichen communities.

**Location:**

European Alps (Austria, Switzerland and Italy).

**Methods:**

Five glacier forelands subjected to relevant glacier retreat during the last century were investigated. In each glacier foreland, three successional stages were selected at increasing distance from the glacier, corresponding to a gradient of time since deglaciation between 25 and 160 years. In each successional stage, soil‐dwelling lichens were surveyed within five 1 × 1 m plots. In addition to a classical ecological framework, based on species richness and composition, we applied a functional approach to better elucidate community assembly mechanisms.

**Results:**

A positive relationship was found between species richness and time since deglaciation indicating that richer lichen communities can be found at increasing terrain ageing. This pattern was associated with compositional shifts, suggesting that different community assemblages can be found along the successional stages. The analysis of β‐diversity revealed a significant nested pattern of species assemblages along the gradient (i.e. earlier successional stages hosted a subset of the species already established in older successional stages), while the turnover component was less relevant. Considering functional groups, we found contrasting patterns in relation to time since deglaciation: the incidence of species with a cyanobacterial photobiont and those reproducing by spores decreased, while that of species reproducing by vegetative propagules increased.

**Main conclusions:**

This study reveals that community assembly patterns of soil‐dwelling lichens in alpine glacier forelands are ruled by mechanisms of directional species accumulation and trait selection that involve a trade‐off between different functional strategies. Functional traits that reflect the dispersal and adaptation capability of the species underpin the colonization success of soil‐dwelling lichens in glacier forelands.

## Introduction

Glacial retreat is accelerating in most mountain regions of the world, and consequently, newly exposed land surfaces are available for biotic colonization (Pepin *et al*., 2015). In this framework, glacier forelands provide an interesting experimental case to better comprehend the spatial‐temporal dynamics of primary biological succession (e.g. Whittaker, 1993; Walther *et al*., 2002; Tscherko *et al*., 2005). Previous studies on plant dynamics indicated a directional pattern of increasing species richness along primary succession that is usually associated with compositional shifts (e.g. Raffl *et al*., 2006; Cannone *et al*., 2008). Most of these studies suggest that community dynamics are generally shaped by two main mechanisms (e.g. Jones & Henry, 2003; Caccianiga *et al*., 2006): (1) a directional replacement starting from species‐poor communities composed of few pioneer species, and (2) a directional species accumulation, leading to a nested community structure along the spatial‐temporal gradient.

A promising avenue to better comprehend community dynamics in primary succession involves the integration of functional traits with traditional approaches (e.g. Walker *et al*., 1986; Huston & Smith, 1987; Chapin *et al*., 1994; Caccianiga *et al*., 2006; Erschbamer & Mayer, 2011). Species functional traits are expected to directly link to environmental factors (Webb *et al*., 2010) providing mechanistic insights on the ecological processes ruling community assembly along environmental gradients (Diaz & Cabido, 2001; De Bello *et al*., 2013; Dainese *et al*., 2015). In particular, variation in species strategies throughout a succession is expected to be explained by a trade‐off between traits (Kneitel & Chase, 2004). For example, Löbel & Rydin (2010) suggested a trade‐off between dispersal and establishment ability on successful colonization of new habitats in bryophytes, indicating that species reproducing asexually have a higher ability to establish, but a lower long‐distance dispersal ability than species with sexual reproduction. Chapin *et al*. (1994) revealed that plant life history traits related to dispersal ability, as well as to nitrogen fixation, were critical to early succession dynamics. Similar results have been recently reported by Erschbamer & Mayer (2011), who elucidated the importance of several functional traits governing the colonization process and the progress of plant succession.

While many studies have focused on the dynamics of vascular plant communities in glacier retreat areas (e.g. Whittaker, 1993; Vetaas, 1994; Tscherko *et al*., 2005; Raffl *et al*., 2006; Cannone *et al*., 2008; Burga *et al*., 2010), a surprising knowledge gap still exists about soil‐dwelling lichens (but see e.g. Favero‐Longo *et al*., 2012). These organisms contribute considerably to the biodiversity of high elevation alpine environments (Nascimbene *et al*., 2012), underpinning relevant ecological functions and ecosystem services (Elbert *et al*., 2012; Zedda & Rambold, 2015). Lichens are a complex symbiotic system based on the interaction between a fungus (mycobiont) and a photosynthetic partner (photobiont), also hosting hyperdiverse microbial communities (Grube *et al*., 2009). The photobiont may improve the capacity of the species to establish and develop in extreme environments like glacier forelands (Haugland & Beatty, 2005), thus facilitating ecological succession (Breen & Lévesque, 2008). In particular, cyanobacterial photobionts can fix atmospheric nitrogen (Rikkinen, 2015) and hence contribute to the biogeochemical cycle of this element that is critical in newly exposed, nutrient‐poor soils (Schmidt *et al*., 2008). Besides the photobiont type, successful dispersal is the precondition for occupying new habitats (Scheidegger & Werth, 2009), such as newly exposed areas after glacier retreat. Lichens have contrasting reproduction strategies, i.e. sexual or asexual reproduction, that influence their dispersal and, consequently, colonization rates (Scheidegger & Werth, 2009; Johansson *et al*., 2012).

Here, we examined communities of soil‐dwelling lichens in five glacier forelands across the European Alps, along a gradient of distance from the glacier edge that reflected a chronosequence of the glacier forelands. In addition to a classical ecological framework, based on species richness and composition analyses, we applied a functional trait approach to better elucidate and interpret community assembly mechanisms. As substrate stability, nutrient availability, and time for colonization increase (Hodkinson *et al*., 2003; Bradley *et al*., 2014), we hypothesize an increasing species richness with succession time. We expect that this pattern would also be associated with compositional shifts according to the hypothesis that sites more recently deglaciated host a subset of the older communities. This should be reflected by a nested community pattern along the chronosequence gradient. Specifically, communities are expected to progressively recruit from a limited pool of effectively dispersed species (Hodkinson *et al*., 2003) that can rapidly reach and adapt under limiting environmental conditions onto newly available habitat patches. This pattern should be related to a trade‐off between dispersal and colonization ability of the species similar to that found in epiphytic bryophyte communities (Löbel & Rydin, 2010). Species with sexual reproduction are assumed to have better long‐distance dispersal and then should have more chances to rapidly reach recently deglaciated moraines (Löbel *et al*., 2006). Instead, species reproducing asexually are assumed to be better adapted to local dispersal suggesting that recently deglaciated moraines are poorly accessible for these species (Löbel *et al*., 2009). Hence, we expect a contrasting relationship between time since deglaciation and the two functional groups. Also, the photobiont may have a key role, mainly in the earlier colonization stages, mediating the adaptation of lichens on such extreme conditions. In this case, we expect that lichens with cyanobacterial photobionts would show a greater contribution to community assembly on more recently deglaciated sites due to their capability to fix atmospheric nitrogen that is crucial in nutrient‐poor, recently deglaciated soils.

## Materials and methods

### Study areas

**Figure 1:**
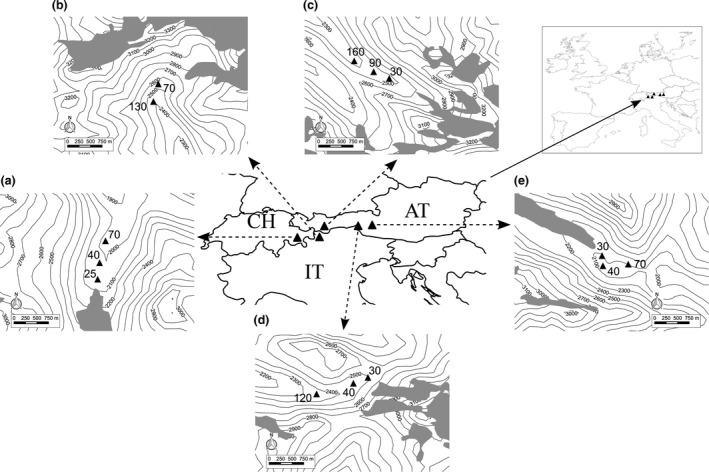
Location of the five glacier forelands distributed across Switzerland (CH), Italy (IT) and Austria (AT) in Central‐Eastern European Alps. For each glacier foreland, the three lichen sampling sites are marked with black triangles and a number indicating the age since deglaciation. Contour lines indicate elevation. Grey areas indicate the glaciers. Glacier forelands: (a) Morteratsch (Switzerland); (b) Matscherferner (Italy); (c) Gaisbergferner (Austria); (d) Rötkees (Italy); (e) Pasterze (Austria).

Five glacier forelands distributed across Switzerland, Italy and Austria (central‐eastern Alps), which have been subjected to glacier retreat during the last century, were investigated (Fig. 1)

*Morteratsch glacier* – this is the third largest and by volume the most massive glacier in the central‐eastern Alps, situated in the Bernina Range in the canton Graubünden, Switzerland. Since 1878, the glacier has retreated almost continuously (average decrease between 1878 and 1998: 16.2 m per year), only interrupted by few periods of slight advances. The climate is continental, the bedrock consists of siliceous rocks, and the soils in the foreland area are weakly developed and have a maximum age of 150 years.

*Matscherferner* – this glacier is situated in the valley “Matschertal” in South Tyrol, Italy. This is a side valley of the Vinschgau (Val Venosta), running from south‐west to north‐east for about 20 km and covering an area of about 100 km^2^. The Matscherferner has retreated continuously in the period between 1927 and 2012 (total decrease of *c*. 570 m; average decrease between 1993 and 2012: 15 m per year). The climate is continental, the bedrock consists of siliceous rocks without calcareous rock, and thus the soils have an acidic character.

*Gaisbergferner* – this is a valley glacier situated in the valley “Gaisbergtal”, which is an alpine side valley of the Gurgler Tal in the Ötztal Alps of Tyrol, Austria. The glacier has retreated almost continuously for the last 70 years, only interrupted by a short period of slight increase in the 1980s (average decrease between 1969 and 2013: 7.2 m per year). The climate is continental, and the dominant rock types mainly include paragneiss, mica‐schists, amphibolites and marble.

*Rötkees* – this glacier is situated in the high valley “Röttal”, which runs from north‐west to south‐east and is a side valley of the Ahrntal (Valle Aurina) in South Tyrol, Italy. The Rötkees has retreated continuously with a total decrease of *c*. 300 m between 1932 and 2011 (average decrease between 1979 and 2011: 9.5 m per year). The climate is continental, and the bedrock of the glacier foreland mainly consists of lime‐containing mica‐schists.

*Pasterze* – with a current length of about 8 km, this is the longest glacier in the eastern Alps, situated within the High Tauern mountain range in Carinthia, Austria. The glacier has retreated almost continuously since the end of the Little Ice Age to 2014 with a total retreat of *c*. 2.2 km, only interrupted by few periods of stagnation or slight advances (average decrease between 1969 and 2013: 21.5 m per year). The Tauern valleys are relatively dry, even if the continental character of the climate becomes weaker with increasing elevation. The dominant rock types mainly include calcareous mica‐schists and green‐schists (= prasinite).

Further information on the study areas can be found in Bilovitz *et al*. (2014a,b,c, 2015a,b).

### Sampling design

In each glacier foreland, three successional stages (sampling sites) were selected at increasing distance from the glacier on the main ground moraine structures, corresponding to a passage of time since deglaciation between 25 and 160 years (Table 1). The first successional stage corresponded to the distance/time since deglaciation at which the first lichen thallus was found. The second stage corresponded to the distance/time since deglaciation at which vegetation is still dominated by cryptogams, while the third stage was placed in areas dominated by vascular plants (grasses and forbs; small trees and shrubs were only present at Morteratsch). In Matscherferner, the stage closest to the glacier was omitted due to logistic constraints. In each successional stage, lichens on soil and plant debris or decaying terricolous bryophytes were surveyed within five 1 × 1 m plots that were selected in the field without any preconceptions on lichen communities. Areas with aquatic habitats and large rocks were avoided. Distance between plots was between 30 and 50 m. In each plot, we recorded the number of 10 × 10 cm subplots in which each species occurred. As local conditions could also contribute to community patterns (Rydgren *et al*., 2014), for each plot we recorded other meaningful predictors, such as elevation, slope, and aspect that were accounted for in the analyses.

**Table 1:** Characteristics of the sampling sites in the five glacier forelands in the central‐eastern Alps, Europe. For each glacier foreland, the number of lichen species, the range of elevation, slope and aspect of the three sampling sites based on values recorded in the field are reported, as well as the distance (m) from the glacier edge and time (years) since deglaciation

Glacier foreland	Number of lichen species	Elevation (m)	Slope (°)	Aspect (°)	Distance from the glacier (m)/time since deglaciation (y)		
					Site 1	Site 2	Site 3
Morteratsch (CH) 46°25′–26′N/09°55′–56''E	15	1965–2020	0–20	0–140	300/25	800/40	1500/70
Matscherferner (IT) 46°46′N/10°41′E	34	2390–2560	5–40	90–180	‐	800/70	1400/130
Gaisbergferner (AT) 46°50′N/11°02′–03′E	41	2350–2460	5–40	0–330	600/30	1000/90	1500/160
Rötkees (IT) 47°01′–02′N/12°10′–11′E	32	2340–2490	7–30	150–310	300/30	600/40	1500/120
Pasterze (AT) 47°04′N/12°44′–45′E	35	2075–2090	0–40	80–300	600/30	1000/40	1300/70

CH, Switzerland; IT, Italy; AT, Austria.

### Species identification and species traits

When possible, lichens were identified in the field. Additionally, in each plot we collected specimens of each species for a more accurate identification in the laboratory. The specimens were identified mainly with the aid of Wirth *et al*. (2013), using routine light microscopy techniques. Some of the identifications required verification using standardized thin‐layer chromatography (TLC), following the protocols of White & James (1985) and Orange *et al*. (2001). The nomenclature mainly follows Wirth *et al*. (2013). Reproductive trait and photobiont type of the species were retrieved from Nimis & Martellos (2008). In particular, species were classified according to their main reproduction strategy as (1) sexually reproducing by spores (size *c*. 10–100 μm), or (2) asexually reproducing by different types of vegetative propagules (size from 50–200 μm to centimetres). Species with cyanobacteria (cyanolichens) included both bipartite species in which cyanobacteria are the main photobiont and tripartite species in which cyanobacteria are secondary photobiont incorporated in specific structures (cephalodia).

### Statistical analyses

#### Species richness

Linear mixed models (LMMs) were used to test the effect of the successional stage (time since deglaciation) and local conditions (elevation, slope, aspect) on plot‐level lichen species richness. Four response variables were tested: (1) total number of species, (2) number of species with cyanobacteria, (3) number of species reproducing by ascospores, and (4) number of species reproducing by vegetative diasporas. The nested sampling design was accounted for in the analysis by including the following random factors: glacier foreland identity and sampling site identity within glacier foreland. LMMs were implemented using the ‘lme4’ package in R (R Development Core Team 2015). We used an information‐theoretic model selection procedure to evaluate alternative competing models using second‐order Akaike's information criterion (AICc) (Burnham & Anderson, 2002). For each parameter, we used model averaging to incorporate model selection uncertainty into our parameter estimates (Burnham & Anderson, 2002). Individual predictor variables that had an Akaike weight > 0.75 or model‐averaged confidence intervals that did not include 0 were considered as the most important predictors. Model comparison was implemented using the ‘MuMIn’ package in R.

#### *Species composition and* β*‐diversity*

We applied canonical ordination in combination with forward selection to test whether the successional stage (time since deglaciation) and local site conditions affect variation in species composition based on species frequencies. For this analysis, we built two matrices: a species‐by‐plot matrix and a plot‐by‐environmental predictors matrix. As a preliminary detrended correspondence analysis (DCA) showed a total inertia 4 standard unit, unimodal ordination (CCA, canonical correspondence analysis) was considered suitable for the data set. To identify which explanatory variables significantly predict species composition, we applied a forward selection based on permutation tests (*n* = 999) and an inclusion threshold of α = 0.05 using the *ordistep* function in the ‘vegan’ package in R. A specific randomization scheme was applied to take into account the hierarchical structure of the sampling design by restricting permutations of samples to each glacier. All the explanatory variables recorded were included in the CCA diagram to provide an overview of the relationships.

We used the method by Baselga (2010) to estimate both the turnover and nestedness‐resultant components of β‐diversity. This method decomposes the overall β‐diversity (measured using the Sørensen dissimilarity index) into two additive fractions describing the species temporal turnover (β_sim_, the dissimilarity due to species replacement) and the variation in species composition due to richness difference in nested patterns (β_nes_). Pairwise β‐diversity measures were calculated using the ‘beta.pair’ function in the ‘betapart'package in R (Baselga & Orme, 2012). Then, we used regression on distance matrices (MRM) (Lichstein, 2007) to examine the relationships between the matrices of turnover and nestedness‐resultant dissimilarities and the Euclidean distance matrix of time since deglaciation (difference in time). We tested both linear and quadratic terms by permutation test (*n* = 9999). The MRM analysis was carried out with the *MRM* function in the ‘ecodist’ package in R (Goslee & Urban, 2007).

Because β_nes_ is a measure of the compositional variation due to nestedness‐resultant dissimilarity rather than a true metric of nestedness (Baselga, 2012), we assessed whether its results were consistent with those provided by NODF (nestedness based on overlap and decreasing fill), an explicit metric of nestedness (Almeida‐Neto *et al*., 2008). NODF is based on the difference between columns (species) and rows (plots) and the paired matching of species occurrences (Almeida‐Neto *et al*., 2008). NODF analysis yields a nestedness score ranging from 0 (non‐nested) to 100 (perfectly nested) and can be computed between species (NODF_species_), sites (NODF_sites_) or both (NODF). We evaluated the statistic using a matrix with the plots (rows) ordered by increasing time since deglaciation and the species ordered by increasing frequencies. We tested the hypothesis that sampling sites more recently deglaciated host a subset of the species already established in sites where glacier retreat occurred over a longer period (‘directional process of species accumulation’), using only the NODF_sites_ on the matrix described above. The significance of the observed nested pattern was tested against 999 null matrices using the null model described in Patterson & Atmar (1986) that maintains observed column totals but allows row totals to vary randomly. This null model preserves species richness per site but allows species composition to vary randomly and equiprobably (Ulrich & Gotelli, 2007). As bedrock type and geographic location may have a strong effect on lichen communities (i.e. the resulting turnover may be larger than that measured in each glacier foreland separately), we repeated the NODF analysis considering the five glaciers separately. NODF analysis was performed using the ‘vegan’ package in R.

#### Functional groups incidence

For each sampling plot, we computed the incidence (% of species) of the three selected functional groups: (1) species with cyanobacteria, (2) species reproducing by ascospores, and (3) species reproducing by vegetative propagules. Then, the incidence of the selected functional groups was used as the response variable in LMMs by including nested random structure (glacier foreland identity and site identity within glacier foreland). The main aim of these analyses was to test whether the proportion of lichens with different dispersal strategy of and cyanolichens changed along the successional stages of the spatial‐temporal gradient. We also accounted for the local site conditions in the models using the same information‐theoretic approach that was applied in the species richness model.

## Results

**Figure 2:**
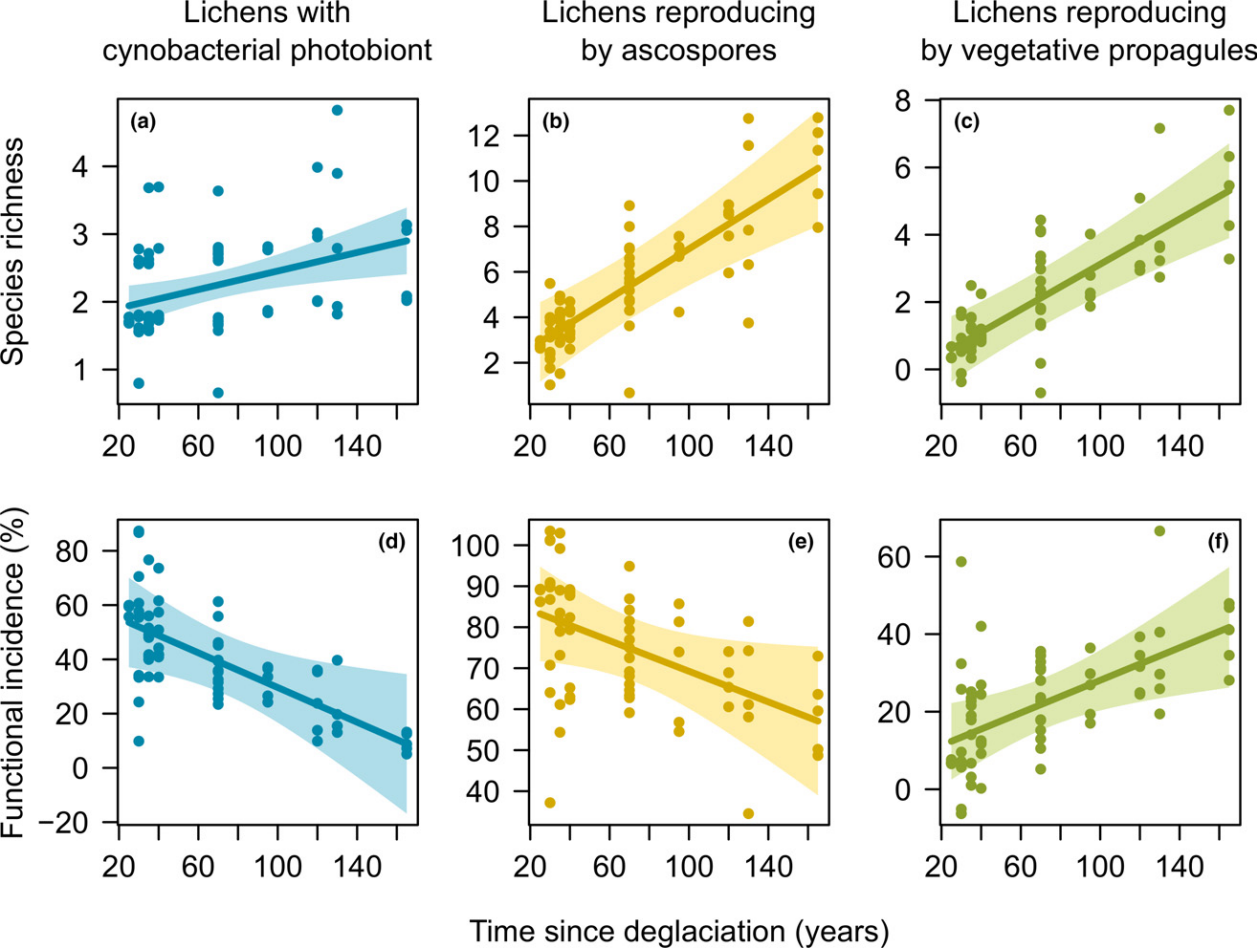
Model estimates and 95% confidence interval from linear mixed models testing time since deglaciation on (a–c) species richness and (d–f) functional group incidence for lichens (a, d) with cynobacterial photobiont, (b, e) reproducing by ascopores, and (c, f) reproducing by vegetative propagules separately, in soil‐dwelling lichen communities in five glacier forelands of the European Alps. The points represent the 70 plots and show the partial residuals from the best plausible model.

In total, 85 lichen species were found (see Appendix S1 in Supporting Information). The total number of lichen species per glacier foreland ranged from 15 to 41 species (Table 1). The average number of species (mean ± SE) in the three successional stage was 7.0 ± 1.8 in earliest stages, 15.6 ± 3.0 in intermediate stages, and 27.2 ± 3.5 in oldest stages. At the plot level, the average number ± SE of species was instead 3.15 ± 0.36 in more recently deglaciated plots, 6.56 ± 0.58 in intermediate plots, and 11.28 ± 0.97 in the oldest plots. Total species richness was best predicted by time since deglaciation (summed Akaike weights, Σ*w*_*i*_ = 1.00), while other factors had low Σ*w*_*i*_ (< 0.60 and 95% CIs including zero) (Table 2). The same result was found for models considering the species richness of functional groups (Table 2). Overall, species richness increased with increasing time since deglaciation for all the functional groups (Fig. 2a–c).

**Table 2:** Standardized model‐averaged regression coefficients (β) and unconditional 95% confidence intervals (CIs) derived from multi‐model inference analysis. The sum of model weights (Σ*w*_*i*_) indicates the relative importance of covariate *i* based on summing weights across the entire model set. Values in bold indicate parameter estimates whose confidence intervals do not overlap zero

	Time		Elevation		Slope		Northness	
	Σ*w*_*i*_	β (CIs)	Σ*w*_*i*_	β (CIs)	Σ*w*_*i*_	β (CIs)	Σ*w*_*i*_	β (CIs)
Species richness								
All species	**1.00**	**0.752 (0.587, 0.917)**	0.25	0.081 (−0.277, 0.439)	0.25	0.029 (−0.136, 0.193)	0.51	0.108 (−0.032, 0.249)
With cynobacteria	**0.88**	**0.302 (0.067, 0.537)**	**0.99**	**0.566 (0.292, 0.840)**	0.23	0.053 (−0.161, 0.267)	0.24	0.022 (−0.175, 0.219)
Reproducing by ascospores	**1.00**	**0.676 (0.390, 0.961)**	0.24	0.097 (−0.384, 0.579)	0.43	−0.127 (−0.289, 0.035)	0.26	0.051 (−0.095, 0.196)
Reproducing by vegetative diasporas	**1.00**	**0.709 (0.466, 0.952)**	0.24	−0.074 (−0.540, 0.391)	0.27	−0.072 (−0.242, 0.098)	0.36	0.091 (−0.056, 0.238)
Functional groups incidence								
With cynobacteria	**1.00**	−**0.497 (**−**0.729, 0.266)**	0.25	−0.065 (−0.398, 0.268)	0.32	−0.106 (−0.337, 0.124)	0.24	0.024 (−0.181, 0.229)
Reproducing by ascospores	**0.93**	−**0.345 (**−**0.593,** −**0.096)**	0.32	0.159 (−0.160, 0.477)	0.24	0.005 (−0.258, 0.268)	0.25	0.042 (−0.185, 0.269)
Reproducing by vegetative diasporas	**0.99**	**0.435 (0.197, 0.673)**	0.36	−0.212 (−0.565, 0.142)	0.24	−0.034 (−0.275, 0.207)	0.27	−0.060 (−0.273, 0.153)

**Figure 3:**
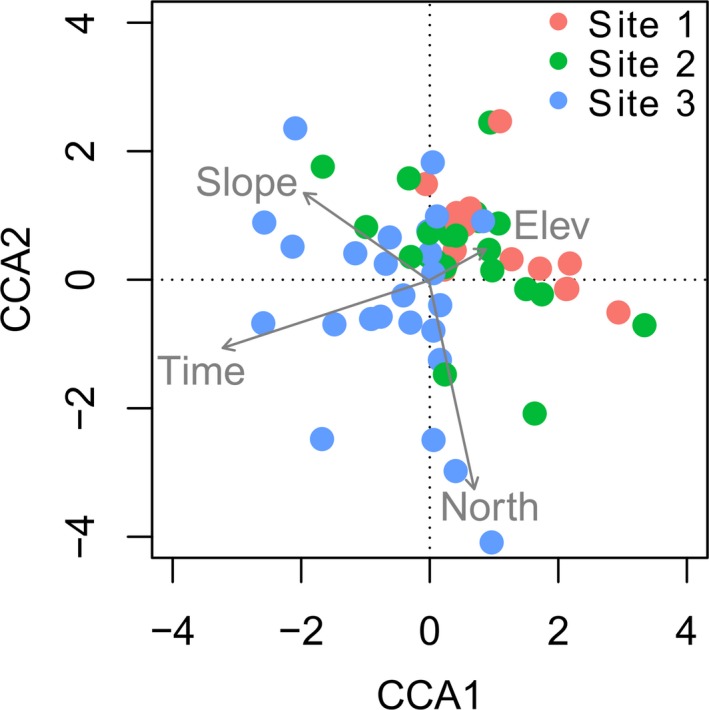
CCA ordination diagram of the 70 plots of soil‐dwelling lichen communities in glacier forelands of the European Alps against the first two canonical axes. Site scores are grouped according to their site position in the five glacier forelands (see Table 1). Axes 1 and 2 explain 44.0% and 27.4% of the total variance, respectively.

**Figure 4:**
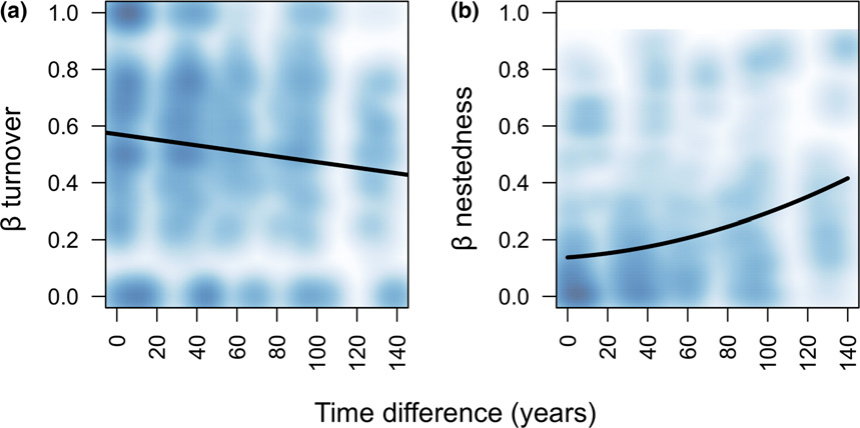
Scatterplot of the relationship for (a) species turnover and (b) nestedness‐resultant dissimilarity versus difference in time since deglaciation in soil‐dwelling lichen communities of five glacier forelands of the European Alps. The fitted line indicates a linear or quadratic regression on distance matrices regression (MRM). The scatterplot is a smoothed shade density representation obtained through a kernel density (function ‘smoothScatter’ in the ‘graphics’ package in R).

The forward selection confirmed that species composition was significantly affected by time since deglaciation (*P* < 0.05) while local site conditions did not show any significant effect (*P* > 0.05). In the CCA ordination diagram, the community assemblages differed along the spatial‐temporal gradient (Fig. 3, Appendix S2). Species turnover (β_sim_) showed a weak negative association with difference in time since deglaciation (MRM, *P* = 0.021, *R* ^2^ = 0.02) (Fig. 4a). On the contrary, we found a strong positive relationship between compositional dissimilarity due to nestedness (β_nes_) and difference in time since deglaciation (MRM, *P* < 0.001, *R* ^2^ = 0.10), i.e. β_nes_ is larger between plots with different successional stages than between plots with similar time since deglaciation (Fig. 4b). Nestedness analysis confirmed a significant nested pattern of species assemblages (NODF_sites_ = 44.106; *P* = 0.001) along the gradient. The same result was found considering the five glaciers separately (Morteratsch, NODF_sites_ = 68.079; *P* = 0.001; Matscherferner, NODF_sites_ = 44.815; *P* = 0.001; Gaisbergferner, NODF_sites_ = 51.798; *P* = 0.001; Rötkees, NODF_sites_ = 41.367; *P* = 0.001; Pasterze, NODF_sites_ = 59.462; *P* = 0.001). This analysis indicated that earlier successional stages host a subset of the species already established in older successional stages. In particular, the average proportion (mean ± SD) of the species found in the earliest stages also occurred in the intermediate (74.5 ± 20%) and oldest stages (75.3 ± 23%) of the same glacier, respectively (differences not significant), while 76 ± 18% of those occurring in the intermediate stages were also found in the oldest ones.

Considering functional groups, all the models were best predicted by time since deglaciation (Σ*w*_*i*_ ≥ 0.90), while local conditions had low Σ*w*_*i*_ (< 0.50) (Table 2). However, we found contrasting patterns in relation to time since deglaciation. The incidence of species with cyanobacterial photobiont and those reproducing by ascospores (good dispersers over long distances) decreased with time since deglaciation (Fig. 2d–e), while that of species reproducing by vegetative propagules (poor dispersers over long distances and good colonization ability) had a positive trend, increasing with time since deglaciation (Fig. 2f).

## Discussion

**Figure 5:**
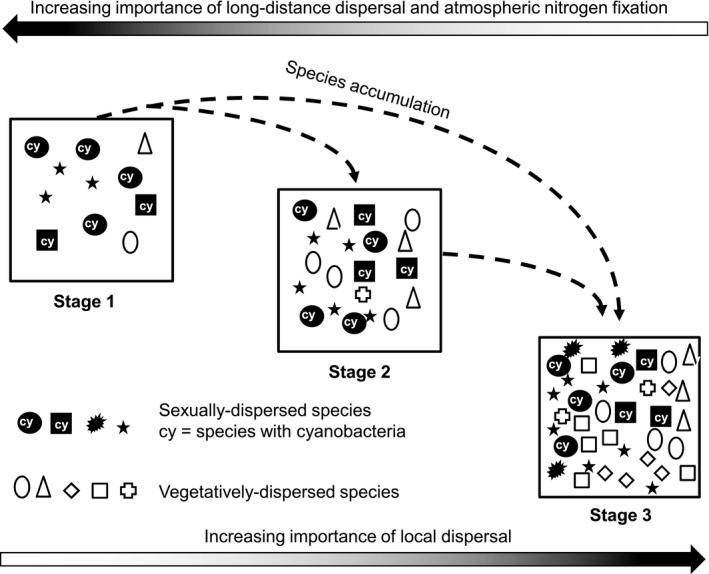
Potential mechanisms that shape the soil‐dwelling lichen assemblages in glacier forelands of the European Alps. Changes of community richness and composition are likely mainly ruled by a directional mechanism of species accumulation reflecting a nested pattern. Our results indicate that species turnover plays only a minor role. The contrasting patterns of spore‐dispersed and vegetatively dispersed species suggest a decreasing importance of dispersal over local recruitment moving from younger to older moraines. On the other hand, such pattern suggests a better establishment ability for species reproducing asexually along the gradient. The pattern of lichens with cyanobacterial photobionts suggests an increasing importance of the nitrogen fixation capacity in younger moraines.

The results confirm our initial hypotheses that community assembly patterns in glacier forelands of the European Alps are mainly ruled by mechanisms of directional species accumulation and trait selection that involve a trade‐off between different functional strategies (Fig. 5). Communities recruit from a pool of effectively dispersed species (Hodkinson *et al*., 2003) that can rapidly reach and colonize recently deglaciated moraines.

### Species richness and composition

We observed a positive species‐time since deglaciation relationship indicating that richer lichen communities can be found at increasing terrain ageing. This relationship is similar to that described in alpine forest ecosystems along a chronosequence of forest age (Nascimbene *et al*., 2010) and likely reflects a combined effect of time per se (i.e. time available for colonization) and chemical–physical changes in substrate conditions associated with terrain ageing (e.g. increased surface availability, substrate stability, nutrient availability). To disentangle the effects of these factors is out of the scope of this study and would require a different design with contrasting substrate conditions at the same age.

As in forest succession (e.g. Nascimbene *et al*., 2010), the increase of species richness along the age gradient is associated with compositional shifts, showing that different community assemblages of soil‐dwelling lichens can be found along the gradient of time since deglaciation. However, our results indicate that community assembly is mainly shaped according to a nested structure along the gradient, in which sites more recently deglaciated host a subset of the species established in sites where glacier retreat occurred over a longer period. The contribution of species turnover to community assembly pattern seems to be less relevant, as indicated by our results on β‐diversity. Species turnover could gain importance when local habitat conditions are more heterogeneous (Nascimbene *et al*., 2013).

### Functional groups

As expected, the analysis of functional groups further fostered a more mechanistic view of community dynamics. While the number of species of each functional group reflects the positive species‐richness time since deglaciation relationship (see previous paragraph), the directional pattern of reproduction strategy and photobiont type incidence along the gradient of time since deglaciation supports the hypothesis that community dynamics are mediated by trait selection and involve both the dispersal capability and the adaptive response of the species to environmental conditions related to glacier retreat dynamics. In particular, the contrasting pattern of the functional groups incidence for spore‐dispersed and vegetatively dispersed species supports the hypothesis of a trade‐off between dispersal and colonization ability also for soil‐dwelling lichens. These patterns suggest a decreasing importance of long‐range dispersal over local recruitment moving from younger to older terrains (Marini *et al*., 2014; Jones *et al*., 2015) and an increasing importance of establishment ability of species along the gradient. The pattern of lichens incidence with cyanobacterial photobionts suggests instead a greater importance of the nitrogen fixation capacity in younger terrains, reflecting an adaptation to extreme substrate conditions.

Considering lichen dispersal capability, our results indicate that species dispersed by spores seem to have more chances to reach recently deglaciated moraines rapidly. Lichens produce thousands of small ascospores (rough length range 10–100 micron) that are easily dispersed. Once the spores arrive on these newly available substrates, the formation of the lichen symbiosis can be sustained by the diverse communities of cyanobacteria and green algae, already established in these primitive environments (Schmidt *et al*., 2008; Frey *et al*., 2013). Conversely, recently deglaciated moraines are poorly accessible for dispersal‐limited species, such as lichen species reproducing by vegetative propagules. This group of lichens is more effective in local dispersal (Löbel *et al*., 2009), given that the relatively small number and the large size of these propagules can limit long‐distance dispersal (Walser, 2004; Scheidegger & Werth, 2009). On the other hand, vegetative propagules seem to have a higher probability of successful colonization than sexual spores (but see Wornik & Grube, 2010) since the photobiont is dispersed with the fungus. Spores arriving on a new substrate must acquire a compatible photobiont to re‐establish the lichen symbiosis. Also, vegetative propagules are probably less adapted to persist in harsh conditions (Nimis & Martellos, 2003), showing a better adaptation to older, more stable sites. These findings highlight the potential for a rapid recruitment of new thalli for species reproducing by vegetative propagules that is likely to allow these species to successfully compete for space with vascular plants in the late phases of the lichen succession.

Considering the photobiont type, our results indicate that lichens with cyanobacteria are particularly adapted to develop viable populations on recently deglaciated sites. Indeed, their capability to fix atmospheric nitrogen, coupled with high rates of carbon fixation (Rikkinen, 2015), emphasises their functional role in acquiring nutrients and, therefore, facilitating ecological succession in glacier forelands (Breen & Lévesque, 2008). An example is the tripartite lichen *Stereocaulon alpinum*, the most abundant species found in our recently deglaciated sites. This species likely plays a key role in transforming the local habitat conditions due to the high rates of metabolic activity of cyanobacterial photobionts encapsulated in external cephalodia (Rikkinen, 2015).

## Conclusions

The nested structure of lichen communities found along our spatial‐temporal gradient corroborates the hypothesis that species accumulation, rather than species replacement, shape lichen community assembly in glacier forelands of the Alps. Moreover, this study reveals that functional groups, reflecting the dispersal and adaptation capability of the species, underpin the colonization success and community assembly patterns of soil‐dwelling lichens in alpine glacier forelands. In particular, a crucial role in colonization dynamics is played by the photobiont, especially in the early stage of establishment, supporting the view that this trait is among the most relevant in mediating the response of lichens to environmental factors (Marini *et al*., 2011; Matos *et al*., 2015). While this study contributes to fill the knowledge gap on primary successions of soil‐dwelling lichens in alpine glacier forelands, we are aware that further research is needed to better clarify community dynamics and the role of species functional traits. For example, similarly to studies on epiphytic lichens (Löbel *et al*., 2006, 2009), a meta‐population approach could help to better elucidate the roles of dispersal and photobionts in shaping lichen dynamics in these environments.

## Biosketch

**Juri Nascimbene** is a plant ecologist with a strong focus on conservation biology in terrestrial ecosystems. He investigates the impact of local and climatic factors on lichen diversity in forest and alpine ecosystems. His research interests in biogeography include the study of lichen diversity patterns along wide environmental gradients.

## Supporting information


**Appendix S1** List of the species.

**Appendix S2** CCA ordination diagram.
