# Supplementary material for: Assembly patterns of soil‐dwelling lichens after glacier retreat in the European Alps
Source: J Biogeogr. 2017 Feb 23;44(6):1393–404. doi: 10.1111/jbi.12970 (PMC5484317; doi:10.1111/jbi.12970)
Supplement: Supplementary file 1 — Appendix S1 List of the species. [file JBI-44-1393-s001.doc]

**Supporting Information**

Juri Nascimbene, Helmut Mayrhofer, Matteo Dainese, Peter Othmar Bilovitz

Assembly patterns of soil-dwelling lichens after glacier retreat in the European Alps

Journal of Biogeography

**Appendix S1.** List of the species.

**Table A1**. List of the species recorded in the five glacier forelands across the Alps. For each species, the total frequency (number of 10x10cm cells occupied) in each glacier foreland is reported as well as the presence of cyanobacteria as photobiont (Cy) and the dispersal type (Sexual vs Vegetative). Detailed floristic data for each site can be found in Bilovitz *et al.* (2014a, 2014b, 2014c, 2015a, 2015b). The specimens are preserved in the herbarium of the Institute of Plant Sciences, University of Graz (GZU). The nomenclature mainly follows Wirth *et al*. (2013).

| Cy | sex/veg | Taxon | Morteratsch | Matscherferner | Gaisbergferner | Rötkees | Pasterze |
| --- | --- | --- | --- | --- | --- | --- | --- |
|  | veg | *Alectoria* *ochroleuca* (Hoffm.) A. Massal. | 0 | 0 | 1 | 0 | 0 |
|  | veg | *Allocetraria* *madreporiformis* (Ach.) Kärnefelt & A. Thell | 0 | 0 | 0 | 1 | 30 |
|  | sex | *Amandinea* *punctata* (Hoffm.) Coppins & Scheid. | 0 | 1 | 0 | 0 | 0 |
|  | veg | *Arthrorhaphis* *citrinella* (Ach.) Poelt | 0 | 3 | 0 | 0 | 0 |
|  | veg | *Arthrorhaphis* spec. [*A*. *alpina* or *A*. *vacillans*] | 0 | 0 | 15 | 0 | 0 |
|  | sex | *Bacidia* *bagliettoana* (A. Massal. & De Not.) Jatta | 0 | 0 | 2 | 0 | 2 |
|  | sex | *Bilimbia* *lobulata* (Sommerf.) Hafellner & Coppins | 0 | 0 | 0 | 0 | 3 |
|  | sex | *Bilimbia* *microcarpa* (Th. Fr.) Th. Fr. | 0 | 0 | 0 | 0 | 0 |
| + | sex | *Blennothallia* *crispa* (Huds.) Otálora, P. M. Jørg. & Wedin | 0 | 0 | 0 | 0 | 4 |
|  | sex | *Bryonora* *castanea* (Hepp) Poelt | 0 | 0 | 1 | 0 | 0 |
|  | sex | *Caloplaca* *ammiospila* (Wahlenb.) H. Olivier | 0 | 9 | 0 | 1 | 0 |
|  | sex | *Caloplaca* *sinapisperma* (Lam. & DC.) Maheu & Gillet | 0 | 0 | 1 | 0 | 0 |
|  | sex | *Caloplaca* *stillicidiorum* s. l. | 0 | 0 | 30 | 4 | 71 |
|  | sex | *Caloplaca* *tiroliensis* Zahlbr. | 0 | 0 | 10 | 0 | 87 |
|  | sex | *Catapyrenium* *cinereum* (Pers.) Körb. | 0 | 0 | 16 | 0 | 0 |
|  | veg | *Cetraria* *ericetorum* Opiz | 3 | 3 | 0 | 2 | 4 |
|  | veg | *Cetraria* *islandica* (L.) Ach. | 129 | 266 | 40 | 44 | 5 |
|  | sex | *Cetraria* *muricata* (Ach.) Eckfeldt | 0 | 101 | 7 | 34 | 20 |
|  | sex | *Cladonia* *amaurocraea* (Flörke) Schaer. | 0 | 0 | 0 | 1 | 0 |
|  | veg | *Cladonia* *arbuscula* s. l. | 2 | 14 | 0 | 0 | 0 |
|  | veg | *Cladonia* *arbuscula* (Wallr.) Flot. subsp. *squarrosa* (Wallr.) Ruoss | 1 | 0 | 0 | 1 | 0 |
|  | veg | *Cladonia* *borealis* S. Stenroos | 1 | 112 | 0 | 0 | 0 |
|  | sex | *Cladonia* *cariosa* s. l. | 18 | 7 | 223 | 82 | 0 |
|  | veg | *Cladonia* *fimbriata* (L.) Fr. | 64 | 0 | 0 | 0 | 0 |
|  | sex | *Cladonia* *gracilis* (L.) Willd. | 2 | 0 | 0 | 0 | 0 |
|  | sex | *Cladonia* *macroceras* (Delise) Hav. | 2 | 41 | 2 | 3 | 2 |
|  | veg | *Cladonia* cf. *mitis* Sandst. | 0 | 121 | 0 | 0 | 0 |
|  | veg | *Cladonia* cf. *pleurota* (Flörke) Schaer. | 0 | 66 | 0 | 0 | 0 |
|  | sex | *Cladonia* *pyxidata* s. l. | 55 | 0 | 99 | 276 | 35 |
|  | veg | *Cladonia* cf. *subulata* (L.) Weber ex F. H. Wigg. | 2 | 0 | 0 | 0 | 0 |
|  | sex | *Cladonia* *symphycarpia* (Flörke) Fr. | 0 | 0 | 141 | 44 | 88 |
|  | sex | *Cladonia* *uncialis* (L.) Weber ex F. H. Wigg. | 0 | 24 | 0 | 0 | 0 |
|  | veg | *Cladonia* spec. | 0 | 21 | 0 | 0 | 0 |
|  | veg | *Dactylina* *ramulosa* (Hook.) Tuck. | 0 | 0 | 4 | 0 | 0 |
|  | sex | *Dibaeis* *baeomyces* (L. f.) Rambold & Hertel | 0 | 6 | 0 | 0 | 0 |
|  | sex | *Diploschistes* *muscorum* (Scop.) R. Sant. | 0 | 0 | 0 | 0 | 5 |
|  | veg | *Flavocetraria* *cucullata* (Bellardi) Kärnefelt & A. Thell | 0 | 0 | 6 | 0 | 2 |
|  | veg | *Flavocetraria* *nivalis* (L.) Kärnefelt & A. Thell | 0 | 174 | 130 | 0 | 21 |
|  | sex | *Fulgensia* *bracteata* (Hoffm.) Räsänen subsp. *deformis* (Erichsen) Poelt | 0 | 0 | 27 | 0 | 124 |
| + | veg | *Fuscopannaria* *praetermissa* (Nyl.) P. M. Jørg. | 0 | 0 | 3 | 0 | 0 |
|  | veg | *Hypogymnia* *phsyodes* (L.) Nyl. | 0 | 0 | 0 | 0 | 3 |
|  | Veg | *Lecanora* *bryopsora* (Doppelb. & Poelt) Hafellner & Türk | 0 | 0 | 2 | 0 | 9 |
|  | sex | *Lecanora* *epibryon* (Ach.) Ach. | 0 | 0 | 0 | 0 | 11 |
|  | sex | *Lecanora* *hagenii* (Ach.) Ach. var. *fallax* Hepp | 0 | 0 | 14 | 0 | 82 |
|  | sex | *Lecidea* *berengeriana* (A. Massal.) Th. Fr. | 0 | 0 | 1 | 0 | 0 |
|  | sex | *Lecidea* *hypnorum* Lib. | 0 | 1 | 0 | 2 | 0 |
|  | sex | *Lecidella* *wulfenii* (Hepp) Körb. | 0 | 0 | 70 | 0 | 1 |
|  | sex | *Lecidoma* *demissum* (Rutstr.) Gotth. Schneid. & Hertel | 0 | 3 | 0 | 0 | 0 |
|  | veg | *Lepraria* *diffusa* (J. R. Laundon) Kukwa | 0 | 10 | 2 | 0 | 0 |
|  | veg | *Lepraria* *eburnea* J. R. Laundon | 0 | 4 | 3 | 0 | 7 |
|  | veg | *Lepraria* *finkii* (Hue) R. C. Harris | 0 | 7 | 0 | 0 | 0 |
|  | sex | *Megaspora* *verrucosa* (Ach.) Hafellner & V. Wirth | 0 | 0 | 5 | 0 | 5 |
|  | sex | *Micarea* *incrassata* Hedl. | 0 | 0 | 0 | 1 | 0 |
|  | veg | *Ochrolechia* *inaequatula* sensu auct. | 0 | 0 | 4 | 0 | 0 |
| + | veg | *Peltigera* *didayctyla* (With.) J. R. Laundon | 0 | 1 | 0 | 0 | 0 |
| + | veg | *Peltigera* *extenuata* (Nyl. ex Vain.) Lojka | 1 | 0 | 0 | 0 | 0 |
| + | veg | *Peltigera* *lepidophora* (Nyl. ex Vain.) Bitter | 0 | 0 | 7 | 11 | 0 |
| + | sex | *Peltigera* *rufescens* (Weiss) Humb. | 0 | 41 | 255 | 29 | 112 |
|  | sex | *Phaeorrhiza* *nimbosa* (Fr.) H. Mayrhofer & Poelt | 0 | 0 | 2 | 0 | 0 |
|  | sex | *Physconia* *muscigena* (Ach.) Poelt | 0 | 0 | 0 | 0 | 45 |
|  | sex | *Placidiopsis* *oreades* Breuss | 0 | 0 | 0 | 0 | 1 |
|  | veg | *Placynthiella* *icmalea* (Ach.) Coppins & P. James | 0 | 0 | 1 | 0 | 0 |
| + | sex | *Protopannaria* *pezizoides* (Weber) P. M. Jørg. & S. Ekman | 0 | 0 | 0 | 37 | 0 |
|  | sex | *Protothelenella* *sphinctrinoidella* (Nyl.) H. Mayrhofer & Poelt | 0 | 0 | 0 | 15 | 0 |
|  | veg | *Pseudevernia* *furfuracea* (L.) Zopf var. *ceratea* (Ach.) D. Hawksw. | 0 | 0 | 0 | 0 | 2 |
|  | sex | *Psoroma* *hypnorum* (Vahl) Gray | 0 | 10 | 0 | 0 | 0 |
|  | sex | *Psoroma* *tenue* Henssen var. *boreale* Henssen | 0 | 5 | 14 | 58 | 0 |
|  | sex | *Pycnothelia* *papillaria* (Ehrh.) Dufour | 0 | 0 | 0 | 0 | 0 |
|  | veg | *Rinodina* *candidogrisea* Hafellner, Muggia & Obermayer | 0 | 0 | 0 | 0 | 3 |
|  | sex | *Rinodina* *mniaraea* (Ach.) Körb. var. *mniaraea* | 0 | 7 | 3 | 2 | 0 |
|  | sex | *Rinodina* *mniaraea* (Ach.) Körb. var. *cinnamomea* Th. Fr. | 0 | 0 | 2 | 0 | 0 |
|  | sex | *Rinodina* *mniaraea* (Ach.) Körb. var. *mniaraeiza* (Nyl.) H. Magn. | 0 | 0 | 0 | 18 | 0 |
|  | sex | *Rinodina* *roscida* (Sommerf.) Arnold | 0 | 0 | 3 | 0 | 1 |
| + | sex | *Scytinium* *intermedium* (Arnold) Otálora, P .M. Jørg. & Wedin | 0 | 0 | 0 | 0 | 2 |
|  | sex | *Solorina* *bispora* Nyl. | 0 | 0 | 16 | 11 | 4 |
| + | sex | *Solorina* *crocea* (L.) Ach. | 0 | 88 | 0 | 0 | 0 |
|  | sex | *Sporodictyon* *terrestre* (Th. Fr.) S. Savić & Tibell | 0 | 0 | 0 | 10 | 0 |
| + | sex | *Stereocaulon* *alpinum* Laurer | 710 | 644 | 919 | 645 | 91 |
| + | veg | *Stereocaulon* *nanodes* Tuck. | 0 | 0 | 0 | 15 | 1 |
|  | sex | *Tetramelas* *insignis* (Nägeli) Kalb | 0 | 1 | 0 | 0 | 0 |
|  | veg | *Thamnolia* *vermicularis* (Sw.) Schaer. var. *vermicularis* | 0 | 154 | 1 | 0 | 17 |
|  | veg | *Thamnolia* *vermicularis* (Sw.) Schaer. var. *subuliformis* (Ehrh.) Schaer. | 0 | 0 | 1 | 0 | 0 |
|  | sex | *Toninia* spec. | 0 | 0 | 10 | 0 | 0 |
|  | sex | *Trapeliopsis* *granulosa* (Hoffm.) Lumbsch | 0 | 3 | 0 | 0 | 0 |
|  | veg | *Vulpicida* *juniperinus* (L.) J.-E. Mattsson & M .J. Lai | 0 | 0 | 0 | 0 | 28 |

**References**

Bilovitz, P.O., *et al.* (2015a) Terricolous lichens in the glacier forefield of the Morteratsch glacier (Eastern Alps, Graubünden, Switzerland). *Phyton*, **55**, 193-199.

Bilovitz, P.O., *et al.* (2015b) Terricolous lichens in the glacier forefield of the Pasterze (Eastern Alps, Carinthia, Austria). *Phyton*, **55**, 201-2014.

Bilovitz, P.O., Nasimbene, J., Tutzer, V., Wallner, A. & Mayrhofer, H. (2014a) Terricolous lichens in the glacier forefield of the Rötkees (Eastern Alps, South Tyrol, Italy). *Phyton*, **54**, 245-250.

Bilovitz, P.O., Tutzer, V., Wallner, A., Nasimbene, J. & Mayrhofer, H. (2014b) Terricolous lichens in the glacier forefield of the Matscherferner (Eastern Alps, South Tyrol, Austria). *Acta ZooBot Austria*, **150/151**, 197-202.

Bilovitz, P.O., Wallner, A., Tutzer, V., Nasimbene, J. & Mayrhofer, H. (2014c) Terricolous lichens in the glacier forefield of the Gaisbergferner (Eastern Alps, Tyrol, Austria). *Phyton*, **54**, 235-243.

Wirth, V., Hauck, M. & Schultz, M. (2013) *Die Flechten Deutschlands*. Ulmer, Stuttgart.
