# Supplementary material for: Assembly patterns of soil‐dwelling lichens after glacier retreat in the European Alps
Source: J Biogeogr. 2017 Feb 23;44(6):1393–404. doi: 10.1111/jbi.12970 (PMC5484317; doi:10.1111/jbi.12970)
Supplement: Supplementary file 2 — Appendix S2 CCA ordination diagram. [file JBI-44-1393-s002.doc]

**Supporting Information**

Juri Nascimbene, Helmut Mayrhofer, Matteo Dainese, Peter Othmar Bilovitz

Assembly patterns of soil-dwelling lichens after glacier retreat in the European Alps

Journal of Biogeography

**Appendix S2.** CCA ordination diagram of the lichen species occurring in the 70 plots against the first two canonical axes. Text will be used for species names with higher priority if labels overlap based on orditorp(vegan) function in R. Species abbreviations: Bry_cas, *Bryonora* *castanea*; Cal_sin, *Caloplaca* *sinapisperma*; Cet_isl, *Cetraria* *islandica*; Cet_mur, *Cetraria* *muricata*; Cla_bor, *Cladonia* *borealis*; Cla_car, *Cladonia* *cariosa*; Cla_mac, *Cladonia* *macroceras*; Cla_mit, *Cladonia* cf. *mitis*; Cla_ple, *Cladonia* cf. *pleurota*; Cla_sp, *Cladonia* spec.; Cla_unc, *Cladonia* *uncialis*; Dac_ram, *Dactylina* *ramulosa* ; Fla_cuc, *Flavocetraria* *cucullata*; Ful_bra, *Fulgensia* *bracteata*; Lec_ber, *Lecidea* *berengeriana*; Lec_dem, *Lecidoma* *demissum*; Lec_epi, *Lecanora* *epibryon*; Lec_wul, *Lecidella* *wulfenii*; Lep_dif, *Lepraria* *diffusa*; Mic_inc, *Micarea* *incrassata*; Pel_did, *Peltigera* *didayctyla*; Phy_mus, *Physconia* *muscigena*; Pro_sph, *Protothelenella* *sphinctrinoidella*; Pyc_pap, *Pycnothelia* *papillaria*; Rin_mnz, *Rinodina* *mniaraea* var. *mniaraeiza*; Ste_alp, *Stereocaulon* *alpinum*; Tet_ins, *Tetramelas* *insignis*; Tha_sub, *Thamnolia* *vermicularis* var. *subuliformis*; Tha_ver, *Thamnolia* *vermicularis* var. *vermicularis*; Ton_sp, *Toninia* spec.; Tra_gra, *Trapeliopsis* *granulosa*; Vul_jun, *Vulpicida* *juniperinus*.
